# Exercise as a mediator of wellbeing

**DOI:** 10.3389/fpsyg.2025.1443397

**Published:** 2025-04-17

**Authors:** Shiya Jiang, Hao Zhang

**Affiliations:** ^1^Physical Education College, Guangdong University of Petrochemical Technology, Maoming, China; ^2^Guangdong University of Petrochemical Technology, School of Automation, Maoming, China

**Keywords:** physical exercise, psychological wellbeing, physical fitness, mediating effect, exercise

## Abstract

**Introduction:**

Recent studies have highlighted physical exercise’s potential impact on college students’ psychological wellbeing. This study aims to explore this relationship further by examining physical fitness’s mediating role.

**Methods:**

A comprehensive questionnaire was distributed to 386 college students, and the collected data were analyzed using advanced statistical techniques to ensure high reliability (*α* = 0.982) and validity (KMO = 0.947).

**Results:**

The results indicated that exercise habits were significantly influenced by the student’s gender and academic major. Furthermore, a notable correlation was observed between physical fitness and psychological wellbeing, with physical fitness also serving as a partial mediator in this relationship.

**Discussion:**

These findings underscore the importance of promoting physical activity within educational institutions to enhance mental and physical health. The mediating role of physical fitness suggests that improvements in physical condition may be essential for realizing the full psychological benefits of exercise. It supports integrating tailored fitness programs into college wellness initiatives to foster overall student wellbeing.

## Introduction

Psychological wellbeing, an important indicator of individuals’ mental health, has been increasingly attracting attention from scholars and the public (Camp et al., 2022; Li and Liang, 2022; Dalky et al., 2022). Psychological wellbeing relates to individuals’ emotional states and directly influences their learning efficiency and social functioning (Xiong et al., 2023). Enhancing psychological wellbeing has become an important research direction in psychology and education (Herbert, 2022; Teede et al., 2023; Lochbaum et al., 2022). In modern society, college students face multiple psychological pressures from academics, job competition, interpersonal relationships, and future planning (Liu et al., 2022). These pressures often impact students’ mental health and quality of life (Lanjekar et al., 2022; Zheng, 2022). However, despite the frequent psychological issues among student populations, many individuals often lack effective coping mechanisms when facing stress (Herbert, 2022; Barbayannis et al., 2022; Saulle et al., 2022). Among numerous possible intervention measures, physical exercise is considered an effective way to enhance mental health due to its low cost and ease of implementation.

Numerous studies have confirmed that physical exercise can significantly improve individuals’ psychological wellbeing and overall health status (Herbert, 2022; Alesi et al., 2022; Verhoeven et al., 2023). Through physical activities, individuals can improve their physical health, alleviate stress, and reduce symptoms of anxiety and depression to some extent (Herbert, 2022; Alesi et al., 2022; Verhoeven et al., 2023). For example, research shows that regular physical exercise can enhance college students’ self-efficacy and social skills, thereby enhancing their psychological wellbeing (Han et al., 2022; Ren et al., 2021). Physical exercise is also seen as an effective stress-coping strategy, as it can enhance mental states by promoting the balance of neurotransmitters such as serotonin and endorphins (Marti-Prats et al., 2023; Boero et al., 2023). The university period is a crucial stage in an individual’s life and a key phase for physical and mental development and stability. Existing research indicates that the depression rate among Chinese college students is 23.8% (National Health Commission of the People’s Republic of China, 2022), highlighting the importance of universities placing a high priority on students’ mental health. Although numerous studies have widely confirmed the positive association between physical exercise and psychological wellbeing, the specific mechanisms of action remain unclear, particularly regarding the mediating role of fitness in the college student population, which has been insufficiently studied.

Researchers have recently focused on various factors influencing psychological wellbeing, with physical fitness being considered an important mediating variable. Improving physical fitness is directly related to better physical health status and may also indirectly enhance psychological wellbeing through enhancing self-worth and a sense of control (Verhoeven et al., 2023; Alarcón-Gómez et al., 2021; Carneiro-Barrera et al., 2022). Furthermore, research has found that gender and the field of study significantly impact psychological wellbeing (Bradley et al., 2000). Students of different genders and majors exhibit variations in their exercise habits and psychological responses. For instance, males generally engage in physical exercise with higher frequency and intensity, while females may excel in dimensions of psychological wellbeing such as self-acceptance and positive interpersonal relationships. These differences may stem from socio-cultural factors, varying individual expectations, or disparities in anticipated salaries after graduation. However, the specific mechanisms by which these gender and major factors interact with physical exercise to influence psychological wellbeing remain to be explored in depth. Therefore, this study will focus on the college student population to investigate the relationships between gender and major differences in the context of physical exercise and psychological wellbeing, analyzing the mediating role of fitness to fill this gap in the existing literature.

This study used a questionnaire survey method to comprehensively collect data from 386 college students to examine how physical exercise enhances their psychological wellbeing through its impact on physical fitness. The questionnaire underwent rigorous reliability and validity testing to ensure the data’s reliability and the analysis’s effectiveness. In the data processing phase, this study employed statistical methods such as independent sample T-tests, variance analysis, Pearson correlation analysis, and structural equation modeling (SEM) analysis using SPSS software. These methodological applications aim to ensure the scientific rigor and accuracy of the research results. At the same time, this study utilizes the Ryff model, which defines students’ psychological wellbeing through six dimensions: autonomy, environmental mastery, personal growth, positive relationships, purpose in life, and self-acceptance (Kishida et al., 2004). This multidimensional framework effectively captures the various aspects of psychological wellbeing and provides a theoretical basis for the analysis in this study. Through these methods, we can explore the direct and indirect relationships between physical exercise, physical fitness, psychological wellbeing, and other potential moderator variables.

The main objective of this study is to investigate how physical exercise influences college students’ psychological wellbeing through improving physical fitness and its underlying mechanisms. By gaining a deeper understanding of the specific impact pathways of physical exercise on psychological wellbeing, this study aims to provide a scientific basis for developing effective mental health intervention strategies. The scientific and clinical significance of the research lies in its contribution to enhancing the understanding and promotion of the importance of physical exercise by higher education institutions and fostering broader support for college students’ physical activities within families and society to enhance their psychological wellbeing and overall health. Additionally, by revealing the influence of gender and major on physical exercise behavior, this study also holds the potential to tailor personalized physical exercise plans for different groups. Ultimately, these research findings can provide theoretical and practical support for enhancing college students’ Psychological Wellbeing, creating a healthier and more positive campus environment.

## Materials and methods

### Research subjects

The study targets college students, and due to the accessibility of the student population at Guangdong University of Petrochemical Technology, we employed a convenience sampling method. First, we determined the survey’s schools, colleges, majors, and classes through convenience sampling. The researchers randomly selected participants from different colleges and majors to ensure diversity. Next, all surveyors received standardized training to ensure consistent survey instructions. Finally, we adopted a method of centralized filling and on-site distribution and collection, distributing a total of 400 questionnaires. All 400 questionnaires were collected, with 14 invalid questionnaires (blank questionnaires deemed not useful) excluded. We utilized a list-wise deletion method to handle missing data, as the number of missing values was small and not expected to significantly impact the reliability of the research results. Additionally, we conducted a missing data analysis and confirmed that the data were missing completely at random (MCAR). Ultimately, there were 386 valid questionnaires, resulting in a response rate of 96.5%.

### Physical exercise behavior questionnaire

Physical Exercise behavior refers to a series of physical activities conducted autonomously by individuals to improve physical fitness, mastering certain sports skills, releasing emotions, and relaxing the body and mind. It includes exercise time, exercise intensity, exercise frequency, as well as exercise programs and forms. The measurement of physical exercise behavior in this study is based on Liang (1994) Physical Activity Classification Scale, which requires students to assess the intensity, duration, and frequency of their participation in physical activities over the past month. A four-point scoring method is used: 1 = never, 2 = rarely, 3 = sometimes, 4 = always, with higher total scores indicating a higher level of engagement in physical exercise behavior.

### Psychological wellbeing questionnaire

The measurement tool for psychological wellbeing used in this study is the version of the “Psychological Wellbeing Questionnaire” designed by Ryff, which consists of 84 items. The questionnaire was translated and back-translated by Professor Wang Xin in collaboration with students who studied in the United States to ensure that each item not only conforms to the language habits of Chinese individuals but also does not change the meaning of the questions. The translated version was provided. The questionnaire has six dimensions and utilizes a six-point scoring method: 1 = strongly disagree, 2 = disagree, 3 = somewhat disagree, 4 = somewhat agree, 5 = agree, 6 = strongly agree. Participants must make a six-level selection based on their own experiences, with higher total scores indicating a higher level of psychological wellbeing (Wu et al., 2019).

### Physical fitness questionnaire

Physical fitness refers to the physical abilities such as strength, speed, endurance, agility, and flexibility that individuals exhibit during physical activities. It is an external manifestation of an individual’s physical strength and weakness, often implicitly reflected in people’s daily lives, studies, and work, as well as in their physical exercise. Measurement of physical fitness in this study involves utilizing a survey questionnaire from the book “Alzheimers-prevention-program-brain-healthy for the Rest of Your Life” by Small and Vorgan (2012). The questionnaire includes three dimensions and requires students to rate their physical fitness (aerobic exercise, strength training, balance, and coordination) over the past 3 months. A three-point scoring method is used: 1 = rarely, 2 = sometimes, 3 = often, with higher total scores indicating a higher level of physical fitness.

### Statistical methods

After excluding disqualified questionnaires, the numbered questionnaires were imported into SPSS 22.0. missing and outlier data were handled by taking the mean. Independent sample t-tests were used to compare differences in gender and academic majors, and analysis of variance was used to compare differences in grades. Additionally, we employed SEM to verify the mediating effects. Specifically, we applied the SEM technique within path analysis to assess the direct and indirect impacts of physical exercise and fitness on mental health.

### Control and verification of common method bias

To control for common method bias, in addition to conducting confirmatory factor analysis and reliability tests on the measurement tools, the Harman single-factor method recommended by Podsakoff was used to test for common method bias. The results indicated that the common method bias in the questionnaire was less than 40%, meeting statistical requirements.

## Results

### The questionnaire demonstrates high reliability and validity

Through reliability analysis, the survey questionnaire designed in this study exhibited good internal consistency and reliability. The questionnaire comprised 114 items divided into four modules: personal information, physical exercise, physical fitness, and psychological wellbeing. The standardized Cronbach’s alpha coefficient for the total score was 0.982, and the internal consistency reliability for each dimension ranged between 0.982 and 0.983. The reliability coefficients of the research data were higher than 0.9, indicating high data reliability quality suitable for further analysis.

Validity analysis was conducted to examine the rationality and meaningfulness of the survey items using factor analysis. Validation was confirmed through indicators such as Kaiser-Meyer-Olkin (KMO) value, communality, variance explained, and factor loading coefficients. The KMO value assesses the suitability of information extraction; commonality helps to eliminate unreasonable research items; variance explained indicates the level of information extraction, and factor loading coefficients measure the relationship between factors (dimensions) and items. The analysis results showed that all research items had communality values higher than 0.4, indicating effective extraction of information from the research items. Additionally, the KMO value was 0.947, exceeding 0.6, suggesting effective information extraction from the research items, which can be utilized for further analysis.

**Figure 1:**
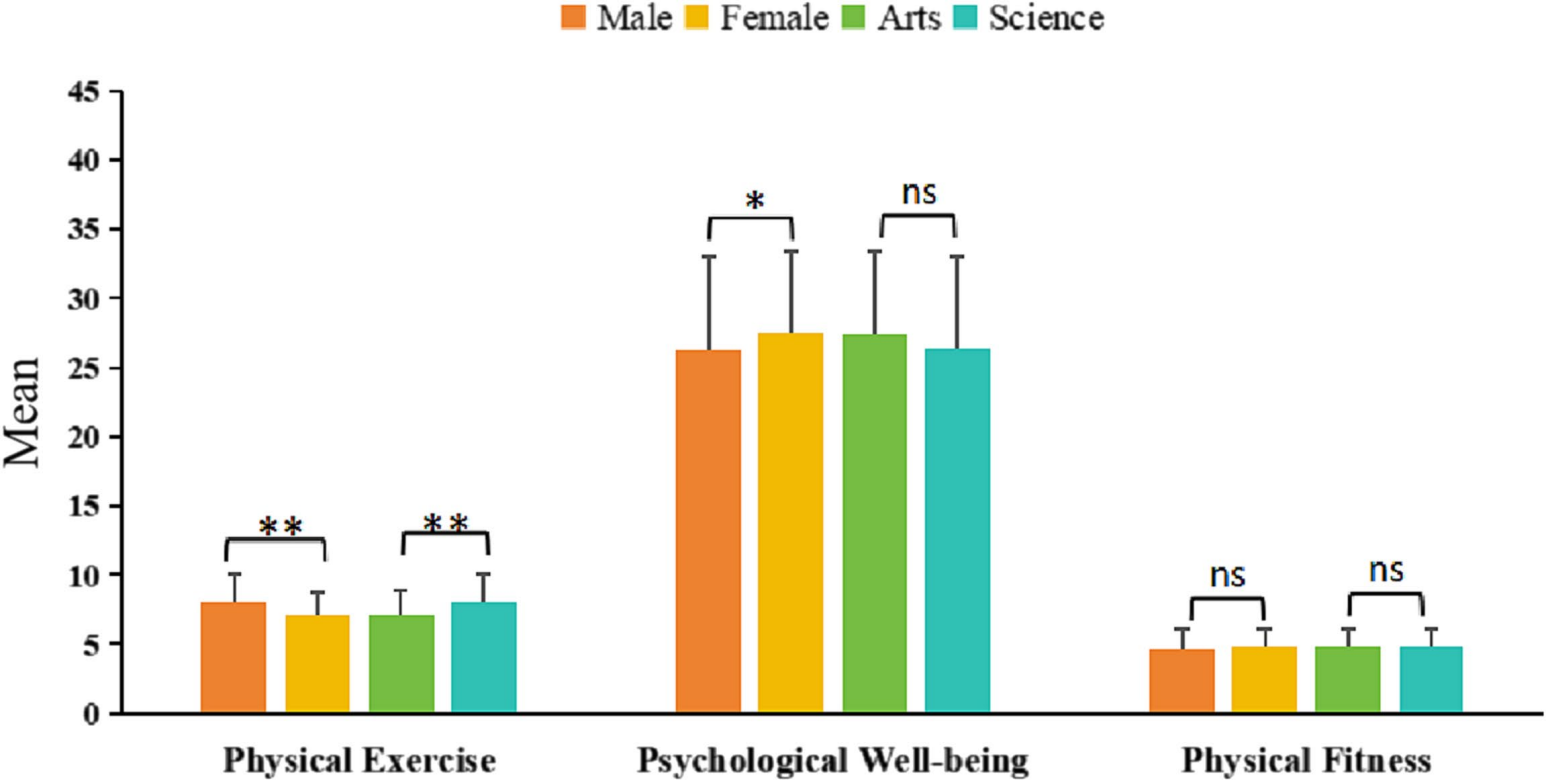
Differentiated analysis of demographic variables. * *p* < 0.05, ** *p* < 0.01, ns, no significance.

Figure 1 shows that according to independent sample T-tests, significant differences were observed in the frequency of physical exercise based on gender (male: 7.99 ± 2.05, female: 7.06 ± 1.75; *p* = 0.000), indicating that males are more active in physical exercise. Additionally, females scored slightly higher than males in psychological wellbeing (female: 27.56 ± 5.83, male: 26.24 ± 6.75; *p* = 0.040), while there was no significant statistical difference between the genders in physical fitness (male: 4.73 ± 1.36, female: 4.84 ± 1.23; *p* = 0.384). The result indicates that gender differences are not a key factor in determining the relationship between physical exercise and wellbeing. Regarding academic background, students in STEM majors scored significantly higher in physical exercise than students in humanities majors (STEM: 7.99 ± 2.03; humanities: 7.11 ± 1.79; p = 0.000), indicating that STEM students engage more frequently in physical activities. However, there was no statistically significant difference in psychological wellbeing between the two disciplines (humanities: 27.41 ± 5.95, STEM: 26.32 ± 6.70; *p* = 0.091). Similarly, there was no significant difference in physical fitness between humanities (4.82 ± 1.26) and STEM (4.75 ± 1.33) students (*p* = 0.592).

**Figure 2:**
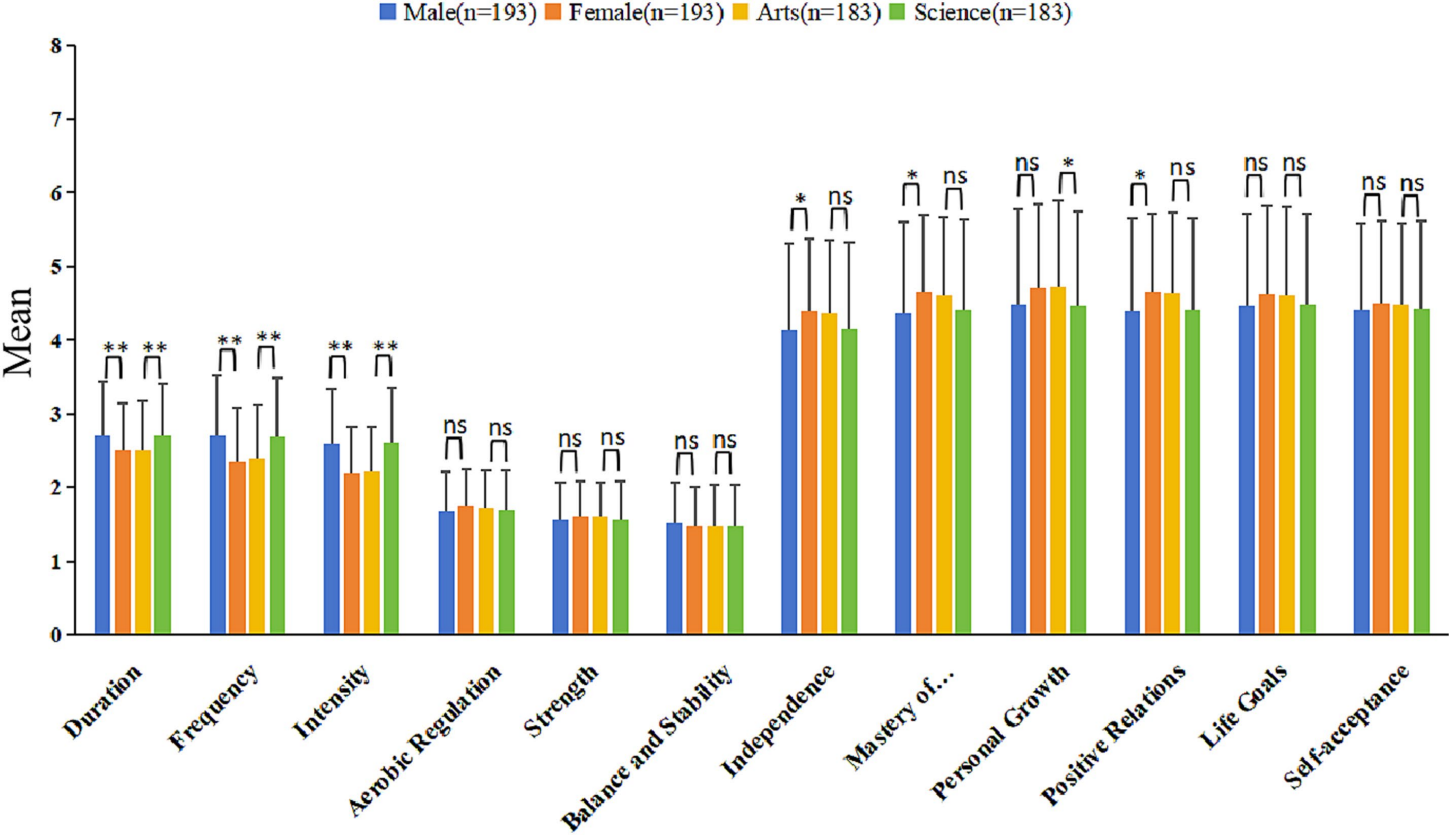
Differentiated analysis of the subdimensions of demographic variables. *p < 0.05, **p < 0.01, ns, no significance.

Figure 2 comprehensively analyzes gender and academic differences in the sub-dimensions of physical exercise, psychological wellbeing, and physical fitness among college students. The results indicate significant differences in all sub-dimensions of physical exercise (including duration, frequency, and intensity) based on gender and academic major. Males and STEM students generally scored higher in these dimensions than females and humanities students. Specifically, males scored higher than females in the sub-dimensions of duration, frequency, and intensity (2.70 ± 0.73, 2.71 ± 0.80, 2.59 ± 0.74 vs. 2.50 ± 0.64, 2.35 ± 0.73, 2.20 ± 0.62). Similarly, STEM students scored higher than humanities students in these dimensions (2.71 ± 0.70, 2.68 ± 0.81, 2.60 ± 0.76 vs. 2.50 ± 0.67, 2.40 ± 0.73, 2.21 ± 0.61).

In the various sub-dimensions of psychological wellbeing (autonomy, environmental mastery, personal growth, positive relationships, purpose in life, and self-acceptance), gender differences were observed in autonomy, environmental mastery, and positive relationships, with females scoring higher than males. Specifically, females scored significantly higher than males in environmental mastery and positive relationships, with scores of 4.66 ± 1.04 and 4.66 ± 1.06, respectively, compared to males with scores of 4.37 ± 1.23 and 4.40 ± 1.25. Furthermore, the dimension of personal growth showed significant differences among different academic majors, with humanities students (4.72 ± 1.17) scoring higher than STEM students (4.46 ± 1.29).

No significant gender or academic major differences were observed in the three sub-dimensions (aerobic regulation, strength, balance, and stability) for physical fitness. This indicates that while there are gender and academic differences in specific behavioral aspects of physical exercise, these differences do not significantly affect overall performance.

### Gender and academic major influence physical exercise and psychological wellbeing

**Figure 3:**
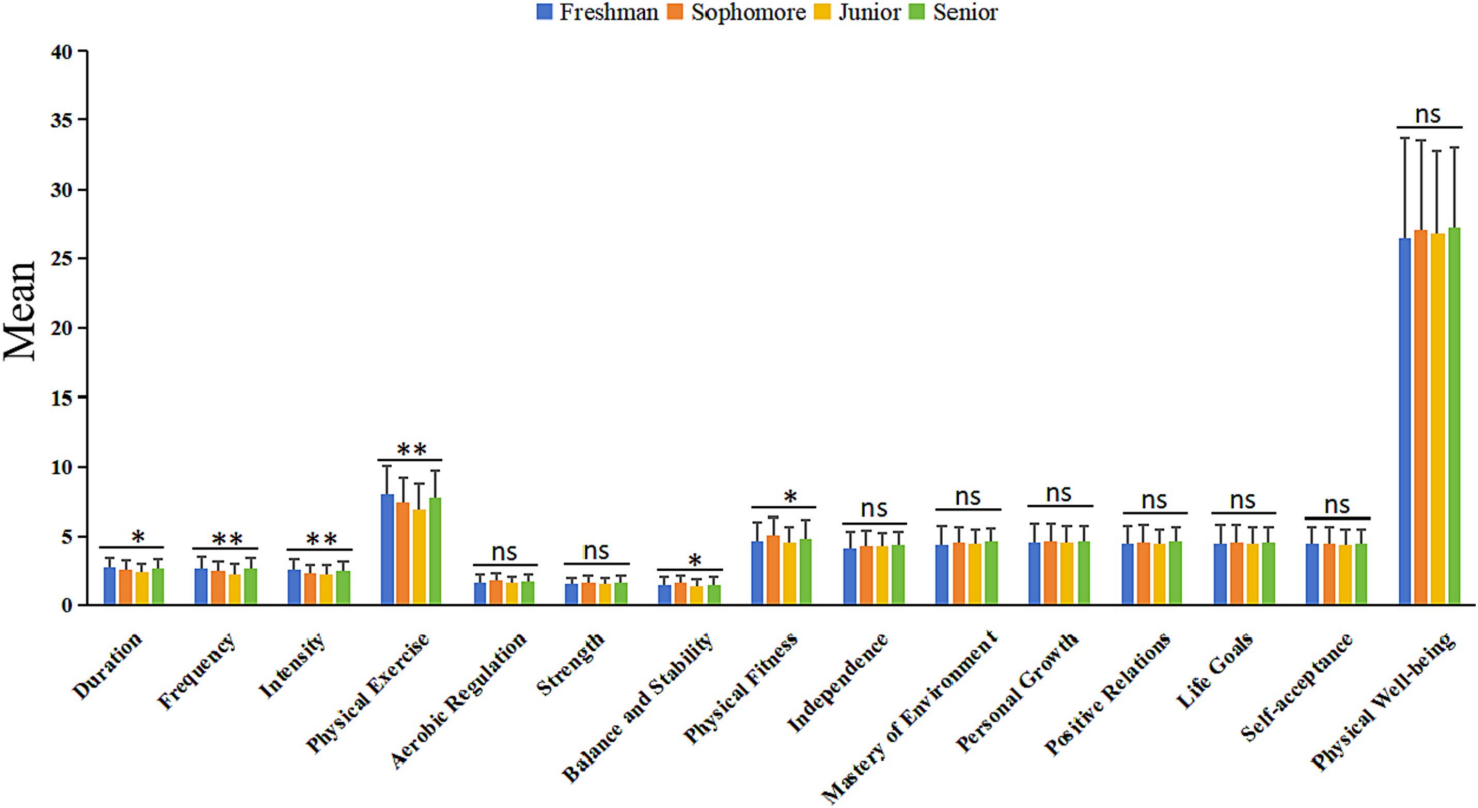
Variance analysis for various subdimensions across four academic years: freshman, sophomore, junior, and senior. *p < 0.05, **p < 0.01, ns, no significance.

Figure 3 provides a detailed analysis of the differences in physical exercise and physical fitness among college students in different academic years while examining how these differences manifest in psychological wellbeing. Through analysis of variance, the research results revealed significant differences in the various sub-dimensions of physical exercise (duration, frequency, intensity, and overall physical exercise score) among different academic years. For example, in terms of the overall physical exercise score, first-year students had the highest average score (7.97 ± 2.09), followed by fourth-year students (7.76 ± 1.94), while third-year students had the lowest score (6.88 ± 1.87). It indicates that as students progress in their academic years, their physical exercise behavior may fluctuate, with higher activity levels in the freshman year potentially decreasing in the middle years and increasing again towards graduation. Similarly, for physical fitness, second-year students had the highest score (5.07 ± 1.27), reflecting that in the mid-years of college, students’ physical fitness may peak. However, this difference was mainly evident in the balance and stability sub-dimension, as there were no significant differences in scores in other sub-dimensions, such as aerobic regulation and strength, indicating that students across different academic years performed relatively uniformly in these aspects of physical fitness.

Although there were significant differences in physical exercise and physical fitness across academic years, the analysis of psychological wellbeing did not show significant differences between the different academic years. This result indicates that while there may be a certain grade-related correlation between physical exercise, physical fitness, and academic years, the direct impact of these variables on psychological wellbeing may be limited, or other factors, such as social support and academic pressure, may have a more significant influence on psychological wellbeing.

### Physical exercise and physical fitness positively predict psychological wellbeing

**Figure 4:**
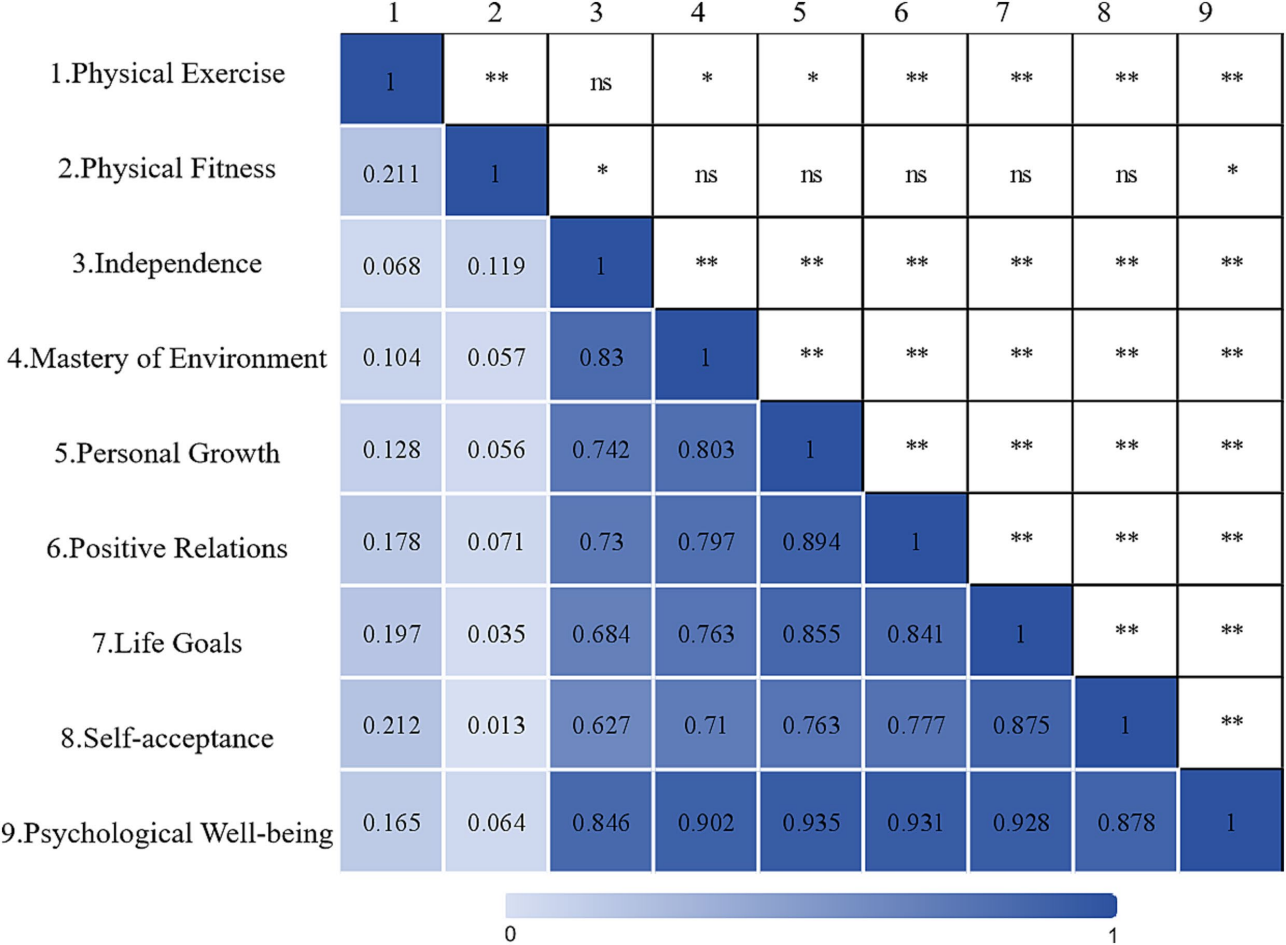
This heatmap illustrates the Pearson correlation coefficients among various variables. *p < 0.05, **p < 0.01, ns, no significance.

Through Pearson correlation analysis, Figure 4 explores the relationships between physical exercise, physical fitness, psychological wellbeing, and their respective sub-dimensions. The analysis results indicate significant positive correlations between physical exercise, physical fitness, and psychological wellbeing, as higher levels of physical exercise are associated with better physical fitness and psychological wellbeing. The correlation coefficient between physical exercise and physical fitness is 0.211, indicating a close relationship between increased physical exercise and improved physical fitness. Furthermore, the correlation coefficient between physical exercise and psychological wellbeing is 0.165, further confirming that physical exercise benefits physical health and positively influences mental health. Additionally, the correlation coefficient between physical fitness and psychological wellbeing is 0.064, indicating a weak but relevant association between physical fitness and psychological wellbeing.

Physical exercise shows varying degrees of positive correlations with the various sub-dimensions of psychological wellbeing, including autonomy, environmental mastery, personal growth, positive relationships, purpose in life, and self-acceptance. The correlation coefficients for these dimensions range from 0.068 to 0.212, indicating that physical exercise may promote psychological wellbeing through multiple pathways. Among these dimensions, the strongest correlation is observed between physical exercise and self-acceptance (0.212), suggesting that physical activities may help individuals better understand and accept themselves.

### Physical exercise partially mediates the effect of physical fitness on psychological wellbeing

**Figure 5:**
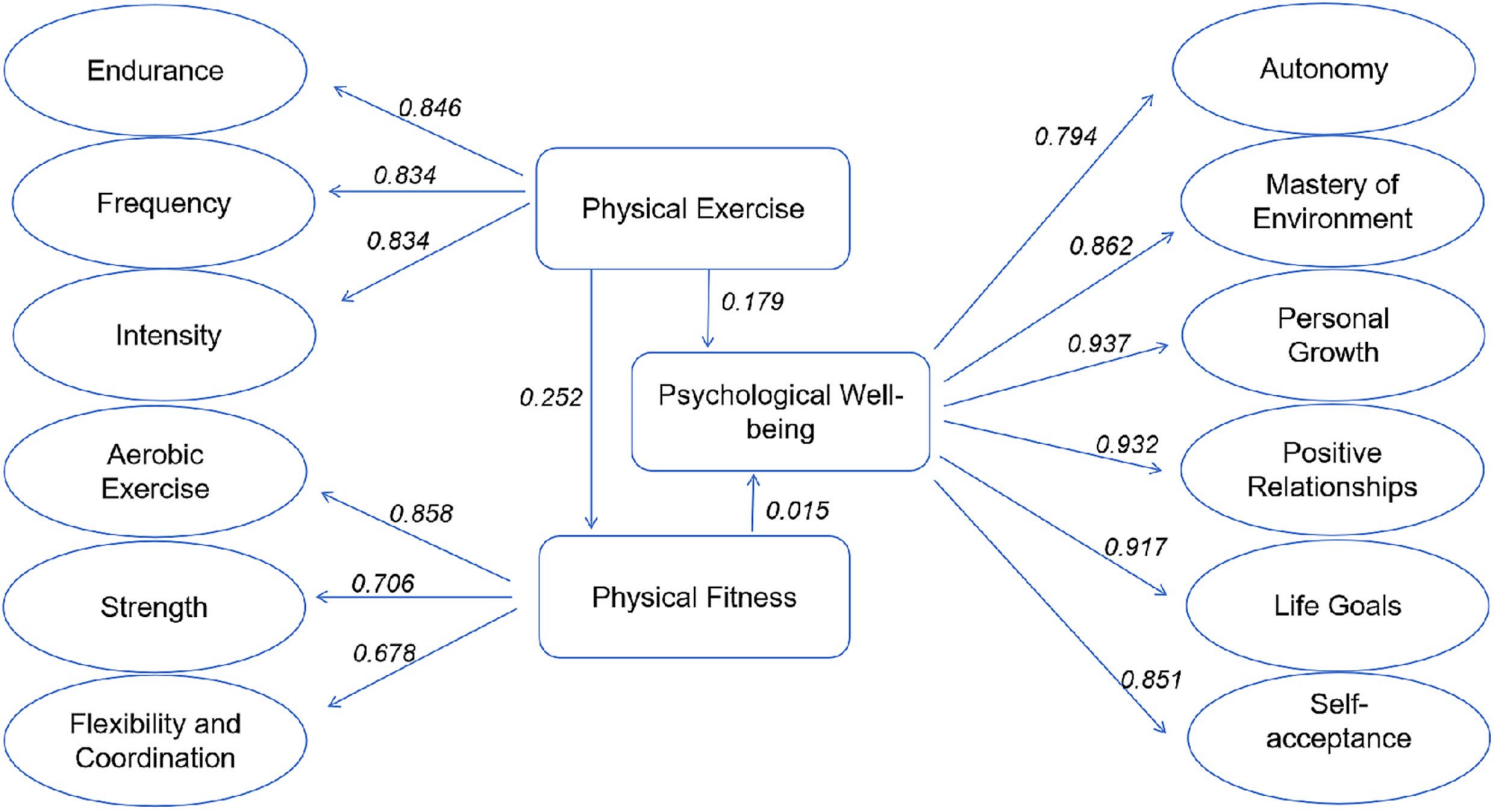
The mediating model of physical fitness between physical exercise and psychological wellbeing of college students.

Before conducting SEM validation, it is essential to establish the independent, dependent, and mediating variables as per Baron and Kenny’s research. Therefore, in this study, physical exercise was set as the independent variable, psychological wellbeing as the dependent variable, and physical fitness as the mediating variable, constructing the hypothetical model (Figure 5). Subsequently, SEM validation was performed using SPSSAU analysis software. The results of the SEM analysis are provided in Table 1 to evaluate the interactions and influence pathways between physical exercise, physical fitness, and psychological wellbeing. The model aims to quantify how physical exercise influences psychological wellbeing through the mediating role of physical fitness via path analysis and their respective direct and indirect effects.

**Table 1:** Estimated parameters table for the structural equation model of physical exercise, physical fitness, and psychological wellbeing.

Variable	Non-standardized parameter estimate	*T*-value	*p*	Standardized parameter estimate	*R*^2^	Direct effect	Indirect effect	Total effect
Physical exercise	0.162	4.118**	0	0.252	0.064	0.252	_	0.252
<-Physical fitness								
Physical exercise	0.267	3.099**	0.002	0.179	0.034	0.179	_	0.179
<-Psychological wellbeing								
Physical fitness	0.034	0.249	0.803	0.015	_	0.015	0.01	0.015
<-Psychological wellbeing								
Model fit indices	χ^2^ = 309.06	df = 66	χ^2^/df = 6.077		RMSEA = 0.716		GFI = 0.879	
	IFI = 0.979	CFI = 0.13	TCI = 0.908		PGFI = 0.574		NFI = 0.917	

χ^2^/df = Chi-square divided by degrees of freedom. RMSEA, Root Mean Square Error of Approximation; GFI, Goodness of Fit Index; IFI, Incremental Fit Index; CFI, Comparative Fit Index; TCI, Tucker’s Congruence Index; PGFI, Parsimony Goodness of Fit Index; NFI, Normed Fit Index. *p < 0.05, **p < 0.01.

In this model, the direct effect of physical exercise on physical fitness showed a significant positive impact, with a non-standardized parameter estimate of 0.162, a standardized estimate of 0.252, and a high level of significance (*p* < 0.01). It indicates that physical exercise significantly enhances physical fitness, reinforcing the theoretical positive impact of physical exercise on physical health. Furthermore, the direct effect of physical exercise on psychological wellbeing was also significant, with a non-standardized parameter estimate of 0.267, a standardized estimate of 0.179, and high significance (p < 0.01). This outcome highlights the direct positive contribution of physical exercise to psychological wellbeing, supporting the importance of physical exercise in promoting mental health. However, the direct effect of physical fitness on psychological wellbeing was not significant (standardized *β* value of 0.015, *p* > 0.05), suggesting that while physical fitness is enhanced by physical exercise, its direct contribution to psychological wellbeing is minimal. Nevertheless, the indirect impact of physical fitness (through physical exercise) is positive, indicating that physical exercise indirectly enhances psychological wellbeing by improving physical fitness.

To evaluate the overall model fit, we analyzed the key model fit indices. As shown in Table 1, the χ^2^/df = 6.077 indicates that the model is relatively complex; the Root Mean Square Error of Approximation (RMSEA) = 0.716 exceeds the conventional standard (< 0.08), suggesting that the model paths may contain some errors and need further optimization. However, the Tucker-Lewis Index (TLI) = 0.908, the Incremental Fit Index (IFI) = 0.979, and the Normed Fit Index (NFI) = 0.917 demonstrate good performance in terms of model parsimony and incremental fit. The Comparative Fit Index (CFI) = 0.13, however, did not meet the recommended threshold of 0.90, indicating that some path relationships within the model may require further refinement. As we could not directly obtain the TLI (Tucker-Lewis Index) during the analysis, we used the IFI and NFI results for supplemental evaluation. These indices indicate that the model exhibits reasonable fit in explaining the relationships between the variables. However, the RMSEA and CFI results suggest that there is still room for improvement in the model structure. Future research should further optimize the model and ensure that all relevant fit indices, including the TLI, are reported to enhance the robustness and credibility of the results. Although we employed the SEM analysis model, we specifically utilized the SEM technique within path analysis to assess the direct and indirect effects of physical exercise and fitness on mental health. We did not conduct gender-based subgroup analyses; however, we plan to explore this in future research.

## Discussion

College students, as a large population, are at a critical age for the onset of mental health issues and should be a focus of concern. With the fast-paced nature of society and the increasingly severe employment situation, college students face pressures from various aspects. Research has found that over one-fifth of college students in China experience varying degrees of psychological distress (Huayang and Xinxin, 2023), leading to a prevalence of mental disorders ranging from 10 to 30%. In an era where mental health issues among college students are prominent, effectively choosing pathways to promote the development of mental health literacy and enhance the overall mental health literacy level of students is essential to achieving the goal of “universal improvement of mental health literacy by 2030″ (Gong and Furnham, 2014).

Self-Determination Theory (SDT) explains how physical activity satisfies basic psychological needs such as autonomy, competence, and relatedness, enhancing mental health. The social significance of SDT is particularly pronounced in the current context of higher education. With societal development and changes in the education system, issues related to students’ motivation and autonomy have become increasingly prominent, directly affecting their academic achievements and mental health (Intrinsic Motivation and Self-Determination in Exercise and Sport).

A systematic review has shown that regular physical activity significantly reduces symptoms of depression and anxiety while enhancing individual psychological wellbeing (Geschlossen oder offen? Ergebnisse operativ versorgter Kalkaneusfrakturen, 2019). This effect may partly result from exercise improving neurophysiological functions, such as increasing serotonin levels in the brain, a key “happiness” neurotransmitter that helps regulate mood and sleep (Shen et al., 2020). Additionally, physical exercise can enhance individuals’ self-efficacy and self-esteem, which are important components of psychological wellbeing (Wang et al., 2019). These physiological and psychological improvements work together, leading individuals engaged in physical exercise to experience higher psychological satisfaction and happiness.

This study reveals the impact of physical exercise on college students’ psychological wellbeing through physical fitness, demonstrating that physical exercise not only directly promotes psychological wellbeing but also indirectly enhances psychological happiness by improving physical fitness. The significance of these findings lies in uncovering the composite benefits of physical exercise on mental health, involving both direct and indirect pathways. The direct positive impact of physical exercise on psychological wellbeing is well-supported by extensive research. This finding is in line with previous research regarding the pathway where physical exercise indirectly enhances psychological wellbeing through physical fitness. Studies suggest that improved physical health indicators, such as higher cardiovascular fitness and lower perception of chronic pain, are positively associated with higher psychological wellbeing (Li et al., 2019). Physical fitness enhancement may increase individuals’ daily functional capabilities, improving social engagement and quality of life, and elevating psychological wellbeing (Faden et al., 2019). It underscores the importance of physical health as a support for mental health, with physical exercise serving as an effective means to achieve this. However, it is worth noting that the direct impact of physical fitness on psychological wellbeing was insignificant in this study, indicating that the influence of improved physical fitness on psychological wellbeing may be more indirect or require additional conditions. For instance, the benefits of physical health may need to be translated into an improvement in psychological wellbeing through improved lifestyle, increased social activities, or higher life satisfaction (Song et al., 2019). It highlights the need for future research to explore additional potential mediator variables, such as social participation and life satisfaction, to comprehensively understand how physical exercise impacts psychological wellbeing through various pathways.

In this study, we explored how physical exercise indirectly affects the psychological wellbeing of college students by improving physical fitness while directly promoting mental health. These findings are generally consistent with previous research but provide new insights at key points. An important study utilized a randomized controlled trial to examine in detail the impact of regular physical exercise on the mental health of adults (Davarpasand, 2019). The study found that individuals engaging in regular physical exercise showed significant improvements in anxiety, depressive symptoms, and overall life satisfaction compared to the control group. It aligns with our findings, indicating that physical exercise can enhance psychological wellbeing. However, our study further revealed the role of physical fitness as a potential mediating variable between physical exercise and psychological wellbeing, offering a deeper understanding of this relationship. Another study explored the physiological mechanisms underlying the relationship between physical exercise and psychological wellbeing, particularly neurobiochemical changes in the brain (Shang et al., 2019). The research indicated that physical exercise directly influences mental state by increasing serotonin levels in the brain and improving overall neural health. These biochemical changes provide a physiological basis for how physical exercise affects psychological wellbeing, which aligns with our study results that state that physical exercise directly impacts mental health.

Additionally, our study considers the social-psychological dimension of this mechanism, suggesting that improving physical fitness can indirectly promote psychological wellbeing, possibly involving broader social activities and self-awareness improvements in individuals. A cross-sectional study involving different populations examined the correlation between the frequency of physical exercise and mental wellbeing (Li et al., 2019). The study found a positive correlation between more frequent physical exercise and higher levels of mental wellbeing, further supporting our finding that physical exercise positively impacts psychological wellbeing.

Based on our research findings, we recommend appropriately increasing the quantity and frequency of physical exercise in intervention programs, advocating for a frequency of three to four sessions per week. This frequency can significantly enhance fitness and improve mental health by increasing physical fitness. Moreover, we believe that interventions should focus on increasing the frequency and duration of exercise and pay special attention to how to effectively enhance physical fitness as a key mediating variable. Designing exercise programs tailored to individuals’ varying fitness levels is particularly important. For example, for those with a lower fitness baseline, starting with low-intensity exercises can gradually build their fitness and increase their motivation to participate. Conversely, increasing the intensity and frequency of exercise should be prioritized for individuals with higher fitness levels to further enhance mental health. Such personalized design can help improve the effectiveness of interventions and ensure long-term participation from individuals.

However, this study did not delve into the specific pathways through which physical exercise affects mental wellbeing, which is an aspect that our research supplements. By introducing physical fitness as a mediating variable, our study not only reveals the direct connection between physical exercise and mental wellbeing but also elucidates how physical exercise indirectly influences mental state through its impact on physical fitness.

In this study, we observed a particular result: although physical exercise significantly improved physical fitness and directly impacted psychological wellbeing, the direct influence of physical fitness on psychological wellbeing did not reach statistical significance. This finding may seem contrary to common belief, as it is generally expected that better physical health should be directly associated with higher psychological wellbeing. However, this specific result may be since psychological wellbeing is influenced by multiple factors, including individual lifestyles, social relationships, psychological coping mechanisms, etc., which may overshadow or diminish the direct impact of physical fitness. For example, even if a person has good physical fitness, negative factors such as insufficient social support or poor coping strategies may offset the positive effects of good physical fitness. Additionally, improving physical fitness may take longer to significantly impact mental states, especially in young college student populations whose psychological changes may be more influenced by immediate psychological and environmental factors (Penedo and Dahn, 2005).

The measurement of physical fitness may not accurately capture the health dimensions closely related to psychological wellbeing. For example, common physical fitness indicators such as strength and endurance may not be as directly related to psychological wellbeing as other health indicators like sleep quality or pain relief. Therefore, future research may need to consider a broader range of physical health indicators to comprehensively assess the impact of physical health on mental states.

Based on these observations and analyses, it can be reasonably inferred that although physical fitness is an important component of psychological wellbeing, its impact on psychological wellbeing may be achieved through other indirect pathways, such as by improving individuals’ physical capacity and social participation, thereby indirectly enhancing psychological wellbeing. Furthermore, this suggests that future health promotion plans should consider adopting multifaceted strategies, focusing on improving physical health and strengthening psychological and social intervention measures to ensure support for psychological wellbeing at multiple levels. Additionally, considering the distinctiveness and diversity of the college student population, future research should further explore the effects of different health dimensions on psychological wellbeing and consider individual differences, such as gender, age, socioeconomic status, etc., to develop more precise and effective health promotion strategies. These measures can help us better understand and utilize physical exercise to enhance psychological wellbeing.

This study explored the mediating effects of physical fitness on college students’ psychological wellbeing through physical exercise, revealing some interesting interactions but also encountering several significant limitations that point toward future research directions. Firstly, one limitation of this study is its cross-sectional design, which cannot establish causality. Although we observed significant correlations between physical exercise, physical fitness, and psychological wellbeing, we cannot determine the causal direction among these variables. For instance, while theoretically, physical exercise may lead to improvements in psychological wellbeing, it is also possible that individuals with better mental states are more likely to engage in physical exercise. Additionally, the study relied on self-reported data, which may be influenced by social desirability bias and memory errors. Self-reported frequency and intensity of physical exercise and quantification of psychological wellbeing may not be as precise as objective measurements, possibly impacting our understanding of the relationships between these variables. In future research, we plan to incorporate objective measures such as fitness testing and psychological health assessments to enhance the accuracy and reliability of our findings.

Secondly, the study sample was limited to college students from a specific region, which may restrict the generalizability of the study results. College students from different regions and cultural backgrounds may vary in their exercise habits, available resources, and the impact of physical exercise on psychological wellbeing. Therefore, the research findings may not easily generalize to other populations or regions. Moreover, although the study attempted to explain the relationship between physical exercise and psychological wellbeing through the mediating effect of physical fitness, it did not explore other potential mediating variables, such as mental resilience, social participation, or stress levels, which could play crucial roles in the relationship between physical exercise and psychological wellbeing. The study is primarily observational and does not incorporate any interventions to assess the impact of specific exercise programs or fitness interventions on mental health. We plan to design future research that includes experimental interventions to further explore these relationships.

Future research should employ longitudinal designs to more accurately investigate the causal relationships between physical exercise, physical fitness, and psychological wellbeing to address these limitations. A more effective analysis of the dynamic relationships and long-term effects between these variables can be conducted by tracking changes in college students’ exercise behaviors and mental states. Additionally, using objective measurement methods (such as activity monitors or psychophysiological measurement techniques) to assess the frequency and intensity of physical exercise and quantify psychological wellbeing through mental assessment tools can help improve the accuracy of data and the reliability of the study. Furthermore, future studies should broaden the sample scope to include college students from different regions and diverse cultural backgrounds, and different age and occupational groups should be considered to explore the universality and cultural specificity of the impact of physical exercise on psychological wellbeing. The research should also consider multiple potential mediating variables to understand how physical exercise influences psychological wellbeing through various pathways.

In conclusion, this study highlighted the significant role of physical exercise in promoting mental health by revealing how it directly and indirectly influences college students’ psychological wellbeing through physical fitness. Despite some limitations, these findings provide valuable insights into the positive impact of physical activity on psychological wellbeing and indicate several directions for future research.

## Conclusion

**Figure 6:**
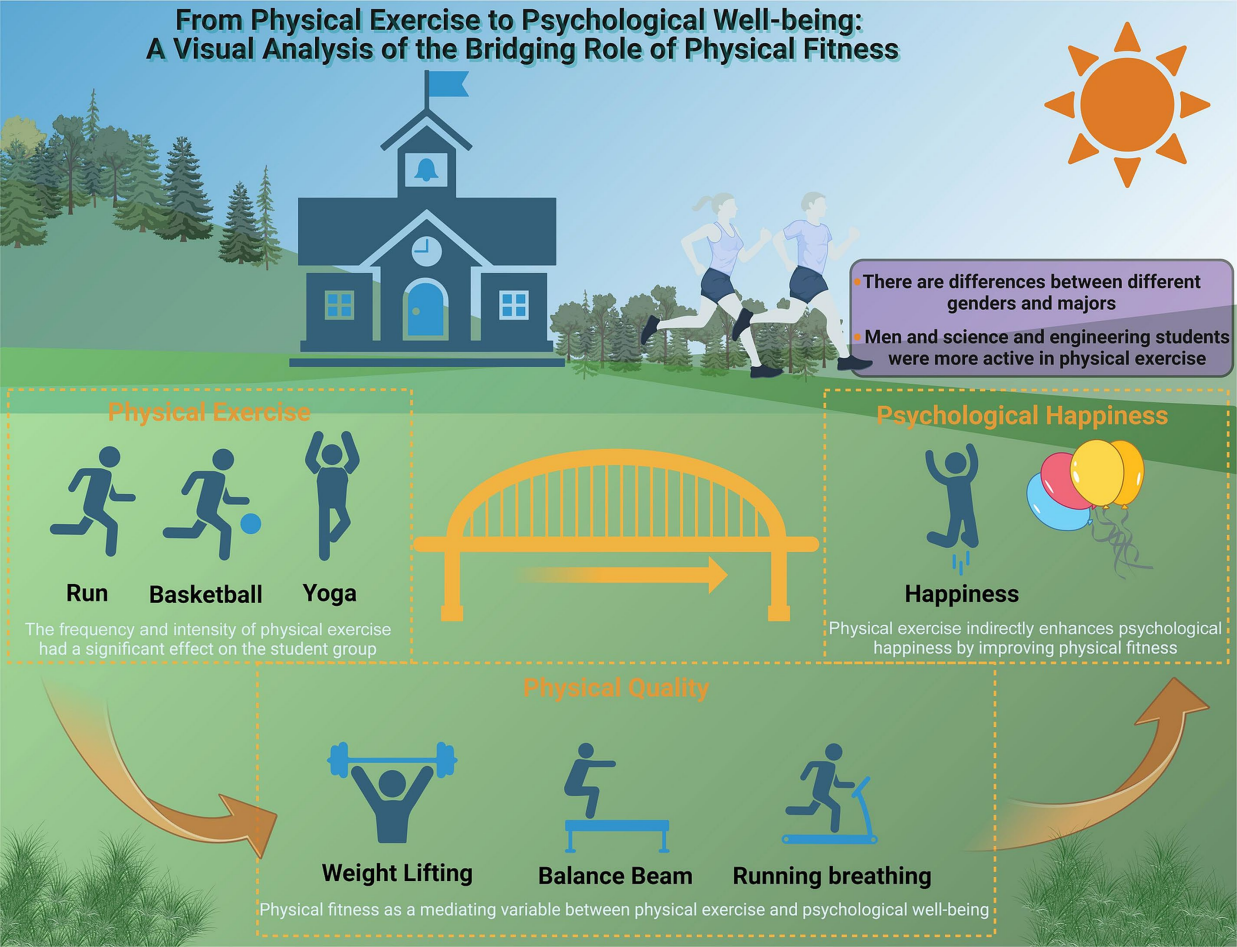
Visual analysis of the mediating role of physical fitness from physical exercise to psychological wellbeing.

This study clarified the positive relationship between physical exercise and college students’ psychological wellbeing and identified that physical fitness partially mediates this relationship (Figure 6). Specifically, physical exercise can directly predict the improvement of psychological wellbeing and indirectly influence psychological wellbeing through improving physical fitness. Additionally, gender and academic major have been confirmed as significant factors influencing physical exercise behavior. It suggests that for college students, physical exercise is not only directly associated with mental health but may also enhance their psychological wellbeing by improving physical fitness.

The scientific value of this study lies in providing a new perspective on how physical exercise influences mental health by emphasizing physical fitness as an important mediating variable. In terms of clinical significance, the research findings can be used to guide schools and health promotion agencies in developing more effective health intervention strategies to promote the psychological wellbeing of young individuals. For example, more personalized physical exercise plans could be developed based on gender and academic major differences.

This study’s limitations include its cross-sectional design, which does not allow for the determination of causality. Additionally, the data was obtained from self-reported questionnaire surveys, which may introduce reporting biases. The study sample was limited to college students, so the generalizability of the results needs further validation.

Future research should employ longitudinal designs to investigate the causal relationships between physical exercise, physical fitness, and psychological wellbeing and consider using objective measurement tools to assess physical exercise and physical fitness. Expanding the sample size to include individuals of different age groups and cultural backgrounds would help further understand the universality of these relationships. Finally, research should explore individual differences, such as personality traits and social support, to understand how they regulate the relationship between physical exercise and psychological wellbeing.

## Data Availability

The original contributions presented in the study are included in the article/supplementary material, further inquiries can be directed to the corresponding author/s.

## References

[ref1] Alarcón-Gómez J. Chulvi-Medrano I. Martin-Rivera F. Calatayud J. (2021). Effect of high-intensity interval training on quality of life, sleep quality, exercise motivation and enjoyment in sedentary people with type 1 diabetes mellitus. Int. J. Environ. Res. Public Health 18:12612. doi: 10.3390/ijerph182312612, PMID: 34886337 PMC8656786

[ref2] Alesi S. Ee C. Moran L. J. Rao V. Mousa A. (2022). Nutritional supplements and complementary therapies in polycystic ovary syndrome. Advances Nutr. 13, 1243–1266. doi: 10.1093/advances/nmab141, PMID: 34970669 PMC9340985

[ref3] Barbayannis G. Bandari M. Zheng X. Baquerizo H. Pecor K. W. Ming X. (2022). Academic stress and mental well-being in college students: correlations, affected groups, and COVID-19. Front. Psychol. 13:886344. doi: 10.3389/fpsyg.2022.886344, PMID: 35677139 PMC9169886

[ref4] Boero G. McFarland M. H. Tyler R. E. O'Buckley T. K. Chéry S. L. Robinson D. L.. (2023). Deleterious interaction between the Neurosteroid (3α,5α)3-Hydroxypregnan-20-one (3α,5α-THP) and the mu-opioid system activation during forced swim stress in rats. Biomol. Ther. 13:1205. doi: 10.3390/biom13081205, PMID: 37627270 PMC10452864

[ref5] Bradley E. H. White W. Anderson E. Mattocks K. Pistell A. (2000). The role of gender in MPH graduates' salaries. J. Health Adm. Educ. 18, 375–389, PMID: 11211353

[ref6] Camp E. E. Sheveleva M. S. Permyakova T. M. Wang K. T. (2022). Family perfectionism among Russian college students. Psychol. Russia 15, 38–55. doi: 10.11621/pir.2022.0303, PMID: 36699135 PMC9833620

[ref7] Carneiro-Barrera A. Amaro-Gahete F. J. Guillén-Riquelme A. Jurado-Fasoli L. Sáez-Roca G. Martín-Carrasco C.. (2022). Effect of an interdisciplinary weight loss and lifestyle intervention on obstructive sleep apnea severity: the INTERAPNEA randomized clinical trial. JAMA Netw. Open 5:e228212. doi: 10.1001/jamanetworkopen.2022.8212, PMID: 35452108 PMC9034401

[ref8] Dalky H. F. Aljawarneh Y. M. Rajab L. M. Almas S. Mazemi F. A. Ali L. A.. (2022). Assessment and evaluation of psychological status of undergraduate college students during COVID-19 pandemic: a cross-sectional study in the United Arab Emirates. Int. J. Environ. Res. Public Health 19:12487. doi: 10.3390/ijerph191912487, PMID: 36231786 PMC9564378

[ref9] Davarpasand T. (2019). Vitamin D deficiency as a seed in a fertile soil: a proposed hypothesis. Echocardiography 36:1019. doi: 10.1111/echo.14343, PMID: 30974008

[ref10] Faden D. L. Ding F. Lin Y. Zhai S. Kuo F. Chan T. A.. (2019). APOBEC mutagenesis is tightly linked to the immune landscape and immunotherapy biomarkers in head and neck squamous cell carcinoma. Oral Oncol. 96, 140–147. doi: 10.1016/j.oraloncology.2019.07.020, PMID: 31422205 PMC7492081

[ref11] Geschlossen oder offen? Ergebnisse operativ versorgter Kalkaneusfrakturen (2019). Zeitschrift fur Orthopadie und Unfallchirurgie 157, 611. doi: 10.1055/a-1009-0046 31794988

[ref12] Gong A. T. Furnham A. (2014). Mental health literacy: public knowledge and beliefs about mental disorders in mainland China. PsyCh J. 3, 144–158. doi: 10.1002/pchj.55, PMID: 26271766

[ref13] Han S. S. Li B. Wang G. X. Ke Y. Z. Meng S. Q. Li Y. X.. (2022). Physical fitness, exercise behaviors, and sense of self-efficacy among college students: a descriptive correlational study. Front. Psychol. 13:932014. doi: 10.3389/fpsyg.2022.932014, PMID: 35910985 PMC9335150

[ref14] Herbert C. (2022). Enhancing mental health, well-being and active lifestyles of university students by means of physical activity and exercise research programs. Front. Public Health 10:849093. doi: 10.3389/fpubh.2022.849093, PMID: 35548074 PMC9082407

[ref15] Huayang K. Xinxin Q. (2023). The positive effects of different exercise modalities on college students’ mental health. Contemp. Sports Technol. 12, 165–168.

[ref16] Kishida Y. Kitamura T. Gatayama R. Matsuoka T. Miura S. Yamabe K. (2004). Ryff's psychological well-being inventory: factorial structure and life history correlates among Japanese university students. Psychol. Rep. 94, 83–103. doi: 10.2466/pr0.94.1.83-103, PMID: 15077752

[ref17] Lanjekar P. D. Joshi S. H. Lanjekar P. D. Wagh V. (2022). The effect of parenting and the parent-child relationship on a Child's cognitive development: a literature review. Cureus 14:e30574. doi: 10.7759/cureus.30574, PMID: 36420245 PMC9678477

[ref18] Li P. Cheng D. Wen J. Ni X. Li X. Xie K.. (2019). The immunophenotyping of different stages of BK virus allograft nephropathy. Ren. Fail. 41, 855–861. doi: 10.1080/0886022X.2019.1617168, PMID: 31535918 PMC6758702

[ref19] Li P. Liang F. (2022). An assessment and analysis model of psychological health of college students based on convolutional neural networks. Comput. Intell. Neurosci. 2022, 7586918–7586910. doi: 10.1155/2022/7586918, PMID: 35785078 PMC9242777

[ref9001] Liang D. (1994). Physical activity rating scale manual (Physical Activity Classification Scale). Echocardiography. Beijing: National Institute of Sports Science.

[ref20] Liu L. Ji Y. Gao Y. Li T. Xu W. (2022). A novel stress state assessment method for college students based on EEG. Comput. Intell. Neurosci. 2022, 4565968–4565911. doi: 10.1155/2022/4565968, PMID: 35712070 PMC9197644

[ref21] Lochbaum M. Stoner E. Hefner T. Cooper S. Lane A. M. Terry P. C. (2022). Sport psychology and performance meta-analyses: a systematic review of the literature. PLoS One 17:e0263408. doi: 10.1371/journal.pone.0263408, PMID: 35171944 PMC8849618

[ref22] Marti-Prats L. Giuliano C. Domi A. Puaud M. Peña-Oliver Y. Fouyssac M.. (2023). The development of compulsive coping behavior depends on dorsolateral striatum dopamine-dependent mechanisms. Mol. Psychiatry 28, 4666–4678. doi: 10.1038/s41380-023-02256-z, PMID: 37770577 PMC10914627

[ref9003] National Health Commission of the People’s Republic of China. Report on National Mental Health Development in China (2021–2022) [in Chinese]. (2022). Beijing: People’s Medical Publishing House.

[ref23] Penedo F. J. Dahn J. R. (2005). Exercise and well-being: a review of mental and physical health benefits associated with physical activity. Curr. Opin. Psychiatry 18, 189–193. doi: 10.1097/00001504-200503000-00013, PMID: 16639173

[ref24] Ren K. Liu X. Feng Y. Li C. Sun D. Qiu K. (2021). The relationship between physical activity and academic procrastination in Chinese college students: the mediating role of self-efficacy. Int. J. Environ. Res. Public Health 18:11468. doi: 10.3390/ijerph182111468, PMID: 34769983 PMC8583502

[ref25] Saulle R. De Sario M. Bena A. Capra P. Culasso M. Davoli M.. (2022). School closures and mental health, wellbeing and health behaviours among children and adolescents during the second COVID-19 wave: a systematic review of the literature. Chiusura della scuola e salute mentale, benessere e comportamenti correlati alla salute in bambini e adolescenti durante la seconda ondata di COVID-19: una revisione sistematica della letteratura. Epidemiol. Prev. 46, 333–352. doi: 10.19191/EP22.5-6.A542.089, PMID: 36384255

[ref26] Shang J. Needleman J. Liu J. Larson E. Stone P. W. (2019). Nurse staffing and healthcare-associated infection, unit-level analysis. J. Nurs. Adm. 49, 260–265. doi: 10.1097/NNA.0000000000000748, PMID: 31008835 PMC6478399

[ref27] Shen C. Wang Z. Zhao F. Yang Y. Li J. Yuan J.. (2020). Treatment of 5 critically ill patients with COVID-19 with convalescent plasma. JAMA 323, 1582–1589. doi: 10.1001/jama.2020.4783, PMID: 32219428 PMC7101507

[ref9002] Small G. W. Vorgan G. (2012). The alzheimer’s prevention program: keep your brain healthy for the rest of your life. New York: Workman Publishing.

[ref28] Song T. Tu M. W. Carnahan C. Cai X. Taniguchi T. Watanabe K.. (2019). Voltage control of a van der Waals spin-filter magnetic tunnel junction. Nano Lett. 19, 915–920. doi: 10.1021/acs.nanolett.8b04160, PMID: 30620202

[ref29] Teede H. J. Tay C. T. Laven J. J. E. Dokras A. Moran L. J. Piltonen T. T.. (2023). Recommendations from the 2023 international evidence-based guideline for the assessment and management of polycystic ovary syndrome. Eur. J. Endocrinol. 189, G43–G64. doi: 10.1093/ejendo/lvad096, PMID: 37580861

[ref30] Verhoeven J. E. Han L. K. M. Lever-van Milligen B. A. Hu M. X. Révész D. Hoogendoorn A. W.. (2023). Antidepressants or running therapy: comparing effects on mental and physical health in patients with depression and anxiety disorders. J. Affect. Disord. 329, 19–29. doi: 10.1016/j.jad.2023.02.064, PMID: 36828150

[ref31] Wang Q. Wang J. Niu S. Wang S. Liu Y. Wang X. (2019). MicroRNA-664 targets paired box protein 6 to inhibit the oncogenicity of pancreatic ductal adenocarcinoma. Int. J. Oncol. 54, 1884–1896. doi: 10.3892/ijo.2019.4759 (Retraction published Int J Oncol. 2022 Aug;61(2):90. doi: 10.3892/ijo.2022.5380), PMID: 30896829

[ref32] Wu J. Lu A. D. Zhang L. P. Zuo Y. X. Jia Y. P. (2019). Zhonghua xue ye xue za zhi =. Zhonghua xueyexue zazhi 40, 52–57. doi: 10.3760/cma.j.issn.0253-2727.2019.01.010, PMID: 30704229 PMC7351698

[ref33] Xiong X. Hu R. X. Chen C. Ning W. (2023). Effects of risk exposure on emotional distress among Chinese adults during the COVID-19 pandemic: the moderating role of disruption of life and perceived controllability. Front. Psych. 14:1147530. doi: 10.3389/fpsyt.2023.1147530, PMID: 37181904 PMC10169736

[ref34] Zheng W. (2022). Cluster analysis algorithm in the analysis of college Students' mental health education. Appl. Bionics Biomechan. 2022:6394707. doi: 10.1155/2022/6394707 PMC9038429 35480710

